# APETALA2-like Floral Homeotic Protein Up-Regulating *FaesAP1_2* Gene Involved in Floral Development in Long-Homostyle Common Buckwheat

**DOI:** 10.3390/ijms25137193

**Published:** 2024-06-29

**Authors:** Qingyu Yang, Lan Luo, Xinyu Jiao, Xiangjian Chen, Yuzhen Liu, Zhixiong Liu

**Affiliations:** College of Horticulture and Gardening, Yangtze University, Jingzhou 434025, China; 2021710827@yangtzeu.edu.cn (Q.Y.); 2022720899@yangtzeu.edu.cn (L.L.); 2021720874@yangtzeu.edu.cn (X.J.); 2023730097@yangtzeu.edu.cn (X.C.); 2023720953@yangtzeu.edu.cn (Y.L.)

**Keywords:** APETALA1, APETALA2, *Fagopyrum esculentum*, floral development, MADS-box

## Abstract

In the rosid species *Arabidopsis thaliana*, the AP2-type AP2 transcription factor (TF) is required for specifying the sepals and petals identities and confers a major A-function to antagonize the C-function in the outer floral whorls. In the asterid species *Petunia*, the AP2-type ROB TFs are required for perianth and pistil development, as well as repressing the B-function together with TOE-type TF BEN. In Long-homostyle (LH) *Fagopyrum esculentum*, VIGS-silencing showed that *FaesAP2* is mainly involved in controlling filament and style length, but *FaesTOE* is mainly involved in regulating filament length and pollen grain development. Both *FaesAP2* (AP2-type) and *FaesTOE* (TOE-type) are redundantly involved in style and/or filament length determination instead of perianth development. However, neither *FaesAP2* nor *FaesTOE* could directly repress the B and/or C class genes in common buckwheat. Moreover, the *FaesAP1_2* silenced flower showed tepal numbers, and filament length decreased obviously. Interestingly, yeast one-hybrid (Y1H) and dual-luciferase reporter (DR) further suggested that *FaesTOE* directly up-regulates *FaesAP1_2* to be involved in filament length determination in LH common buckwheat. Moreover, the knockdown of *FaesTOE* expression could result in expression down-regulation of the directly target *FaesAP1_2* in the *FaesTOE*-silenced LH plants. Our findings uncover a stamen development pathway in common buckwheat and offer deeper insight into the functional evolution of AP2 orthologs in the early-diverging core eudicots.

## 1. Introduction

The ABC model of flower development explains how three classes of homeotic genes confer identity to the four types of floral organs in *Arabidopsis thaliana* [1,2]. According to this model, *APETALA2* (*AP2*) and *AGAMOUS* (*AG*) represent A- and C-class genes that act in an antagonistic fashion to specify the perianth (sepal and petal) and reproductive organs (stamen and pistil), respectively. AP2 binds the AT-rich target sequence lying at the *AG* second intron and directly restricts *AG* expression in the sepals and petals [3]. Moreover, AP2 also antagonizes AG activity in the center of the flower to regulate floral stem cells and maintains shoot apical meristem (SAM) activity in part by keeping *WUSCHEL* (*WUS*) expression active [4,5]. As a flowering repressor, AP2 is negatively regulated by miR172 and MADS-box transcription factor FRUITFULL (FUL), as well as directly repressing the expression of flowering-promoting transcript *AP1* to control the timing of the floral transition [6,7,8]. In addition, the *AP2* orthologs (*BrAP2a* and *BnAP2*) from *Brassica* Crops showed high conservation in specifying sepal development [9]. Losses in the function of *BrAP2a* or *BnAP2* will result in a sepal–carpel modification phenotype. The AP2 family can be divided into the euANT, basalANT, and euAP2 lineages [10]. The euAP2 lineage can be further divided into two different classes called the TOE and AP2 types [11]. Within the euAP2 clade, *AP2* controls flower development and floral organ identity, while *TOE1* and *TOE3* (*TARGET OF EAT1/3*) regulate flowering [12,13]. However, in the asterid species *Petunia* (*Petunia hybrida*), the TOE-type gene *BLIND ENHANCER* (*BEN*) acts as a floral repressor and represses the C-function together with BLIND (BL), while the AP2-type *REPRESSOR OF B-FUNCTION* (*ROB*) genes are required for perianth and pistil development, as well as repress the B-function (but not the C-function) together with *BEN* in the first floral whorl [11]. The *BEN* and *ROB* act as major regulators of nectary size [14]. In rice, *OsMADS1* and miR172s/AP2s formed a regulatory network involved in regulating the elongation of the lemma and the palea (homologous to the eudicot sepal) [15]. In orchids, the *Cymbidium ensifolium AP2*-like gene *CeAP2* shows an antagonistic expression pattern with *AG* ortholog and is negatively regulated by miR172 [16], which suggests a conservation regulatory network of *AG* ortholog and miR172s/*CeAP2*. Previous studies suggested that euAP2 genes were recruited to ovule, fruit, and floral organ development during angiosperm evolution, and changes in the role of *euAP2/TOE3* homologs during development are most likely due to changes in regulatory regions [17].

**Figure 1:**
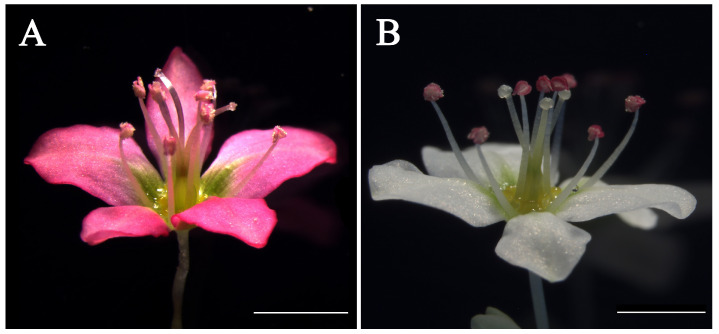
Long-homostyle flower of *Fagopyrum esculentum*. (**A**) Red LH flower with five tepals, eight long stamens, and three long styles; (**B**) white LH flower with five tepals, eight long stamens, and three long styles. Scale bar = 2 mm.

Common buckwheat (*Fagopyrum esculentum*) (Polygonaceae) belongs to the order Caryophyllales (an early-diverging core eudicots clade) and has distylous flowers with single-whorl showy undifferentiated perianth (tepals), representing an obvious difference with most core eudicots flowers [18,19]. Moreover, heteromorphic self-incompatibility (HSI) due to its distylous flowers (pin and thrum) results in successful mating only between flowers with different morphs, which makes it a major obstacle for establishing pure lines, fixation of agronomically useful genes, and genomics-assisted breeding of common buckwheat [20,21]. Here, we have developed a self-compatible (SC) common buckwheat line with uniform long homostyle (LH) flowers (Figure 1) that could freely outcross with pin, thrum, and LH plants [22]. Hence, the LH common buckwheat can easily be the fixation of useful agronomical traits, which also makes it an excellent candidate line to explore the molecular mechanism of floral development and evolution in the buckwheat genus. Here, we separately cloned a TOE-type gene *FaesTOE*, an AP2-type gene *FaesAP2,* and an *AP1* orthologous gene *FaesAP1_2* from LH common buckwheat. Yeast one-hybrid (Y1H) assays were performed to screen whether *FaesTOE* or *FaesAP2* could directly regulate ABC MADS-box genes of common buckwheat to control floral development and floral organ identity. Seven common buckwheat ABC MADS-box gene promotors and a *FaesELF3* gene promotor [*pFaesAP1_1* (MK327554.1); *pFaesAP1_2*; *pFaesAP3_1* (MK956946.1); *pFaesAP3_2* (MN016952.1); *pFaesPI_1* (OM032614.1); *pFaesPI_2* (OM032615.1); *pFaesAG* and *pFaesELF3* (OP572282.1)] containing AT-rich target sequences that we isolated before were screened [19,22,23,24,25]. Only *FaesTOE* can directly bind *pFaesAP1_2*. The dual-luciferase reporter (DR) and VIGS assays further suggested that *FaesTOE* directly activates *FaesAP1_2* (but not the C-functional gene *FaesAG*) to regulate filament length. Moreover, VIGS-silencing LH common buckwheat showed that *FaesAP2* is mainly involved in controlling filament and style length, but *FaesTOE* is mainly involved in regulating filament length and pollen grain development. Both *FaesTOE* and *FaesAP2* genes share redundant functions in regulating filament length determination in LH common buckwheat. Our results also extend the genetic framework for studying floral diversity in the early-diverging core eudicots and offer deeper insight into the evolutionary history of the AP2 family.

## 2. Results

### 2.1. Isolation and Sequence Analysis of APETALA2 Homologous Genes, FaesAP1_2, and Its Promoter from Long-Homostyle Common Buckwheat

**Figure 2:**
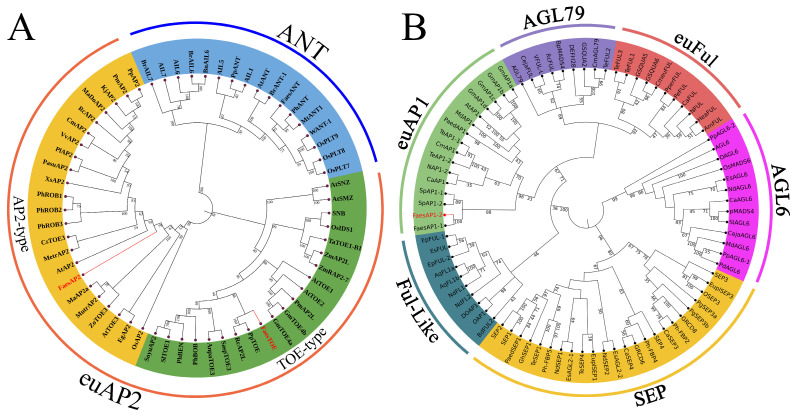
Phylogenetic tree of AP1-Like and AP2-like proteins. (**A**) Phylogenetic tree of *FaesAP2*, *FaesTOE,* and other AP2-Like proteins from different species; (**B**) phylogenetic tree of *FaesAP1_2* with AP1/SEP/AGL6 proteins of other different species. Common buckwheat *FaesAP2*, *FaesTOE,* and *FaesAP1_2* proteins have been marked in red text. AP2-like proteins and AP1/SEP/AGL6-like proteins from other species are separately listed in Supplementary Tables S2 and S3. The numbers represent the Bootstrap percentage values calculated by 1000 replicates.

Two *AP2* homologous genes, *FaesAP2* and *FaesTOE*, were isolated from LH common buckwheat. The 1668 bp *FaesAP2* cDNA contains a 1374 bp ORF (Open Reading Frame, ORF) encoding 457 amino acids (aa) (KM386628.1). The 1286 bp *FaesTOE* cDNA contains a 1047 bp ORF encoding 348aa (PP357007). Phylogenetic tree analysis grouped the FaesAP2 and FaesTOE into the euAP2 lineage (Figure 2A). Moreover, the *FaesAP2* gene was an ortholog of *Arabidopsis AP2* and was designated as *FaesAP2* (*Fagopyrum esculentum AP2*), while the *FaesTOE* gene was an ortholog of *Arabidopsis TARGET OF EAT1* (*TOE1*) and was named *FaesTOE* (*Fagopyrum esculentum TOE1*).

An *AP1* homologous gene, *FaesAP1_2*, and its promoter (*pFaesAP1_2*) were also isolated from LH common buckwheat. The 1026 bp *FaesAP1_2* cDNA contains a 750 bp ORF encoding 249aa (PP357009). Phylogenetic tree analysis grouped the *FaesAP1_2* into the euAP1 lineage (Figure 2B) and revealed that the *FaesAP1_2* shared the same evolution branch with another common buckwheat AP1-like protein, *FaesAP1_1* (AKI81897.1). The gene was an ortholog of *Arabidopsis AP1* and was designated as *FaesAP1_2* (*Fagopyrum esculentum AP1_2*).

The 662 bp *FaesAP1_2* promoter (*pFaesAP1_2*) fragment (−593/+69) was cloned from LH common buckwheat, and the putative transcription start site and cis-acting regulatory elements of the *pFaesAP1_2* were shown in Figure S1. The *pFaesAP1_2* contains a CCAATBOX1 for CONSTANS protein binding to regulate flowering [26]. The *pFaesAP1_2* also contains three GTGANTG10-boxes and four POLLEN1LELAT52-boxes, which are usually found in the promoter region of stamen-development genes [27,28]. Moreover, the *pFaesAP1_2* promoter contains a CArG-box for recognition and binding by floral homeotic MADS-box transcription factors [29]. In addition, *pFaesAP1_2* has two key AACAAA-/TTTGTT-motifs (−557/−552 and −128/−123) for floral homeotic AP2-like protein recognition and action [3]. Moreover, a MYBPLANT motif for flower-specific MYB protein recognition and binding was also found in the *pFaesAP1_2* region [30]. These cis-acting elements indicated that *FaesAP1_2* may be activated by the upstream transcription factors and be involved in the regulation of flowering and/or floral organ development.

In addition, there is a PYRIMIDINEBOXOSRAMY1A-box [31] and a DRECRTCOREAT motif [32,33] lying at the *pFaesAP1_2* region, which suggests that the *FaesAP1_2* expression may also be induced by stress (such as drought, high salt, cold, etc.) and Gibberellin (GA) levels.

### 2.2. FaesTOE Up-Regulate FaesAP1_2 Involved in Floral Organ Development

**Figure 3:**
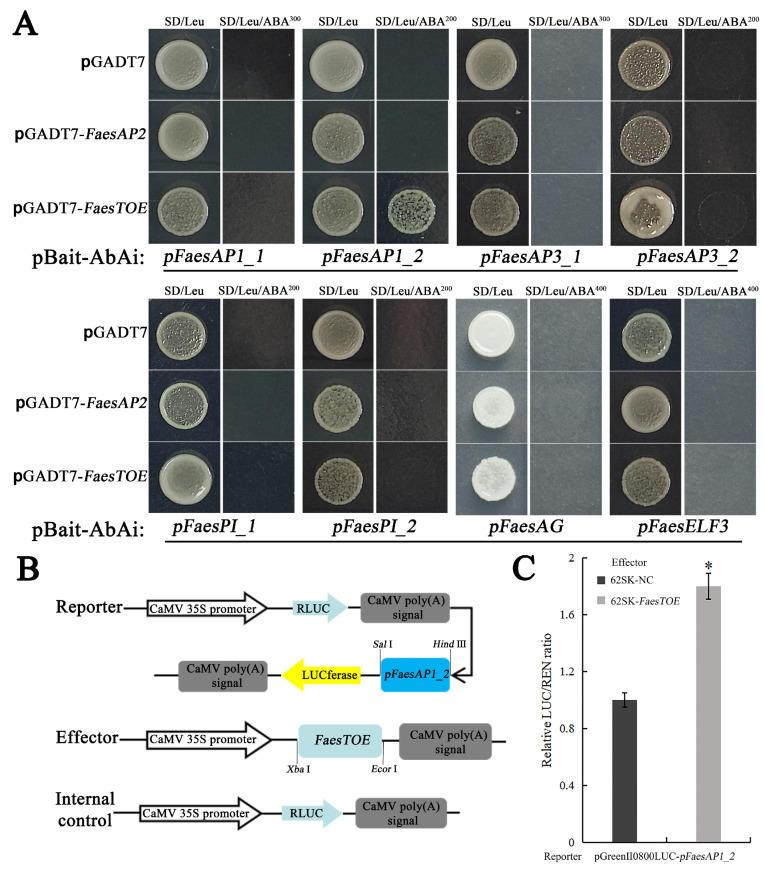
Positive regulation of *FaesAP1-2* by transcription factor *FaesTOE*. (**A**) Y1H screened promoters that could be bound by the *FaesTOE* and/or *FaesAP2* transcription factors. pGADT7 vector was used as a negative control; (**B**) diagram of the reporter and effector constructs; (**C**) results of relative luciferase activity (LUC/REN) in *Nicotiana benthamiana* leaves. Asterisks (*) indicates a statistical significance at *p* < 0.05.

Previous studies showed that the floral homeotic protein AP2 transcription factor could bind the AACAAA-/TTTGTT-motifs and participate in the regulation of flower development [3]. In order to assay whether *FaesAP2* and/or *FaesTOE* directly regulate floral homeotic MADS-box gene or not, seven MADS-box gene promotor regions containing AACAAA-/TTTGTT-motifs, such as *pFaesAP1_1* (−1004/−999, and −921/−916), *pFaesAP1_2* (−128/−123), *pFaesAP3_1* (−620/−615), *pFaesAP3_2* (+117/+122), *pFaesPI_1* (−884/−879), *pFaesPI_2* (−329/−324, and +56/+61), and *pFaesAG* (−879/−874, and −718/−713) were screened. In addition, an *EARLY FLOWRING3* homologue promoter *pFaesELF3* containing AACAAA-/TTTGTT-motifs (+93/+98, +194/+199, +320/+325, +529/+534) was also detected. Yeast one-hybrid (Y1H) assays showed that only *FaesTOE* could directly bind the target fragment (AACAAA-/TTTGTT-motif) of *pFaesAP1_2* (Figure 3A). In addition, the dual-luciferase reporter assays (DR) further showed that *FaesTOE* up-regulates *FaesAP1_2* in plants (Figure 3C). When the reporter construct carried the *pFaesAP1_2* (−450/+35) promoter region, the ratio of LUC/REN expression was significantly increased (*p* < 0.05) (Figure 3B,C).

### 2.3. Expression Analysis of FaesAP2 and FaesTOE in LH Flower F. esculentum

**Figure 4:**
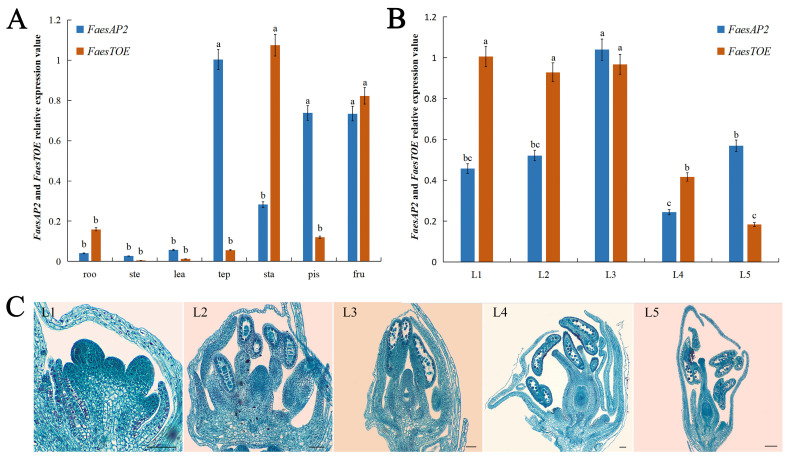
*FaesAP2* and *FaesTOE* expression in different organs and at the sequential development stage of floral buds in LH *F. esculentum*. (**A**) *FaesAP2* and *FaesTOE* expressions in the root (roo), stem (ste), juvenile leaf (lea), tepal (tep), stamen (sta), pistil (pis), and 6-day-old fruit (fru) were detected by qRT-PCR; (**B**) *FaesAP2* and *FaesTOE* expressions were detected by qRT-PCR at sequential development stages of floral buds. (**C**) Cytomorphological section of LH floral buds at sequential development stages; L1: pistil and stamen primodium appearance; L2: filament rapid elongation and anther at microspores tetrads stage; L3: anther at mononuclear microspore; L4: style elongation and mature pollen appearance; L5: maturity floral bud with mature pollen before bloom. Scale bar: (L1, L2, L3, L4, L5) 100 μm. Different letters indicate a significant difference (*p* < 0.05, *LSD*).

*FaesAP2* was mainly expressed in all flower organs (tepal, stamen, pistil) and young fruit of the LH *F. esculentum* (Figure 4A), while *FaesTOE* was mainly expressed in root, stamen, pistil, and young fruit. In addition, the expression level of *FaesAP2* in the tepal or pistil was significantly higher than that of the stamen (*p* < 0.05, *LSD*), while the expression level of *FaesTOE* in the stamen was significantly higher than that of the other floral organ (tepal or pistil) (*p* < 0.05, *LSD*) (Figure 4A). However, the expression level of *FaesAP2* in pistil was significantly higher than the expression level of *FaesTOE* in pistil (*p* < 0.05, *LSD*), while the expression level of *FaesTOE* in stamen was significantly higher than the expression level of *FaesAP2* in stamen (*p* < 0.05, *LSD*) (Figure 4A). Obvious expressions of *FaesAP2* and *FaesTOE* were detected when pistils and stamens emerged in LH floral buds (Figure 4B,C). In addition, *FaesAP2* expression increased constantly during the filament’s rapid elongating stage and achieved its peak until anthers at the mononuclear microspore stage (Figure 4B,C—L2, L3). Then, the *FaesAP2* expression began to drop sharply at mature pollen appearance (*p* < 0.05, *LSD*) (Figure 4B,C—L4). However, *FaesTOE* expression is maintained at a high level until anther at the mononuclear microspore stage (Figure 4B,C—L1, L2, L3) and begins to drop as the mature pollen appearance (Figure 4B,C—L4).

### 2.4. Characterization of FaesAP2, FaesTOE and FaesAP1_2-Silenced Plants

**Figure 5:**
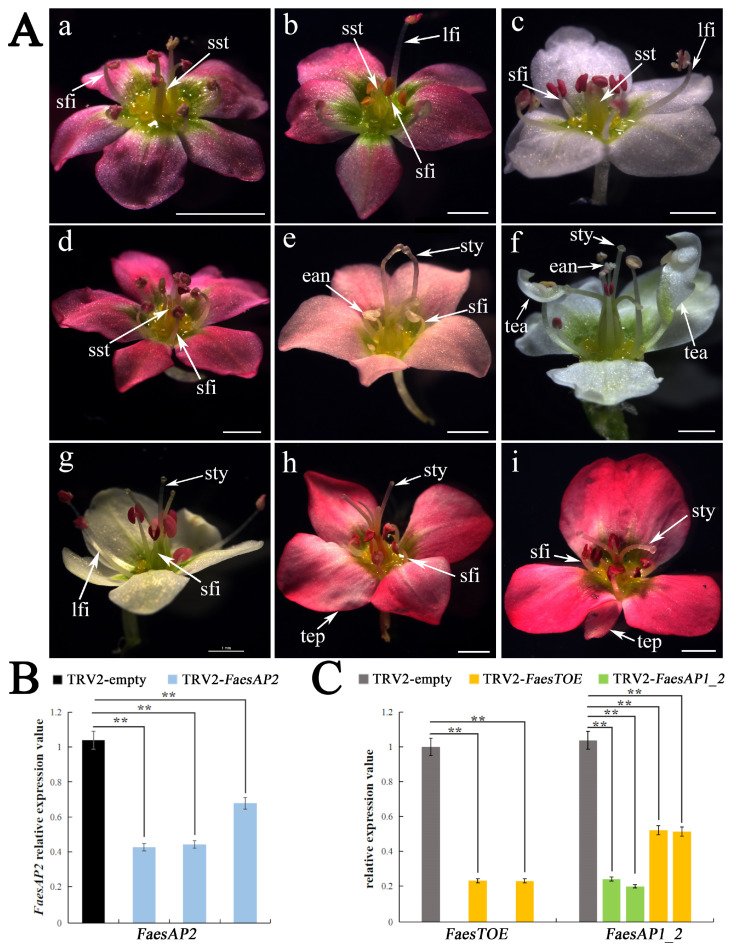
Phenotypes and gene expression of *FaesAP2*, *FaesTOE,* and *FaesAP1_2*-silenced LH *F. esculentum* flowers. (**A**): Phenotypes of *FaesAP2*, *FaesTOE*, and *FaesAP1_2*-silenced LH flowers. (**Aa**) TRV2-*FaesAP2*-treated red flower with three short styles and eight short stamens; (**Ab**) TRV2-*FaesAP2*-treated red flower with three short styles, five short stamens, and three long stamens; (**Ac**) TRV2-*FaesAP2*-treated white flower with three short styles, five short stamens, and three long stamens; (**Ad**) TRV2-*FaesTOE*-treated red flower with three short styles and eight short stamens; (**Ae**) TRV2-*FaesTOE*-treated red flower with three long styles and seven short stamens consisting of short filaments and empty anthers (male sterile anther); (**Af**) TRV2-*FaesTOE*-treated white flower with three tepals, two filaments with anthers homeotically converted into tepaloid structure (tepal attachment of male sterile anther) on the top, three filaments with sterile anthers on the top, three long stamens, and three long styles; (**Ag**) TRV2-*FaesAP1_2*-treated white flower with three long styles, three long stamens, and five short stamens; (**Ah**) TRV2-*FaesAP1_2*-treated red flower with four long styles, six short stamens, and four tepals; (**Ai**) TRV2-*FaesAP1_2*-treated red flower with two long styles and six short stamens, four tepals. Style (sty), tepal (tep), long filament (lfi), short filament (sfi), short style (sst), tepal attachment of anther (tea), empty anther (ean); scale bar = 1 mm; double asterisk (**) indicates statistical significance at *p*-value ≤ 0.01. (**B**) The expression levels of *FaesAP2* in the TRV2-empty-treated and TRV2-*FaesAP2*-treated LH flowers with strong phenotypic changes; (**C**) The expression levels of *FaesTOE* and *FaesAP1_2* in the TRV2-empty-treated, TRV2-*FaesTOE*-treated, and TRV2-*FaesAP1_2*-treated LH flowers with strong phenotypic changes. The grey column was TRV2-empty-treated LH flowers, the green column was TRV2-*FaesAP1_2*-treated LH flowers, and the orange column was TRV2-*FaesTOE*-treated LH flowers.

In order to explore the functions of *FaesAP2*, *FaesTOE,* and *FaesAP1_2*, a virus-induced gene silencing (VIGS) technique via the Tobacco Rattle Virus (TRV) system was performed to knock down their expression in red and white flower LH *F. esculentum,* respectively. Eight TRV2-*FaesAP2*-treated, ten TRV2-*FaesTOE*-treated, and six TRV2-*FaesAP1_2*-treated LH plants producing flowers with the biggest phenotypic changes were obtained, respectively. Both strongly affected TRV2-*FaesAP2*-treated and TRV2-*FaesTOE*-treated red flower LH plants produced flowers with style- and filament-length decreased obviously (Figure 5(Aa,Ad)). Moreover, some VIGS-*FaesAP2* treatment LH flowers have obvious short styles and part of short stamens with anthers at around the same height as the stigmas (Figure 5(Ab,Ac)). However, some VIGS-*FaesTOE* treatment LH flowers have normal long styles but abnormal short stamens consisting of short filaments and empty anthers (male sterile anther) (Figure 5(Ae)), or flowers have normal long styles but part abnormal tepals and stamens, which displayed part stamens with anther homeotically converted into tepaloid structure or sterile anther on the top (Figure 5(Af)). In addition, strongly affected TRV2-*FaesAP1_2*-treated white LH flowers have normal styles but part abnormal short stamens with an obvious reduction in filament length (Figure 5(Ag)). Moreover, some TRV2-*FaesAP1_2* treatment red LH flowers showed tepal numbers and filament length decreased, and long style numbers changed (Figure 5(Ah,Ai)).

In addition, the expression levels of *FaesAP2*, *FaesTOE,* and *FaesAP1_2* were separately detected by qRT-PCR in the gene-silenced plants. The results showed that *FaesTOE* expression was significantly decreased in the flowers of *FaesTOE*-silenced LH plants (Figure 5C, orange column). However, the *FaesAP1_2* expression was significantly decreased in both the flowers of *FaesAP1_2*-silenced (Figure 5C green column) and *FaesTOE*-silenced LH plants (Figure 5C orange column) (*p* < 0.01, *LSD*). All these suggested that the knockdown of *FaesTOE* expression could result in expression down-regulation of the directly target *FaesAP1_2* in the *FaesTOE*-silenced LH plants (Figure 5C).

## 3. Discussion

In the rosid species *A. thaliana*, *AP2* confers A functions and represses expression of C-class gene *AG* in the out two floral whorls to specify perianth (sepal and petal) identity, as well as directly represses the expression of flowering promoting transcripts *AP1* to control the timing of the floral transition [3,6,7,8], while *TOE1* and *TOE3* (*TARGET OF EAT1/3*) regulate flowering [12,13]. Both *AP2* and *TOE* are negatively regulated by miR172 [7]. In *Brassica* Crops (relative species of *A. thaliana*), the *AP2*-like genes (*BrAP2a* and *BnAP2*) play a key role in sepal modification [9]. Losses in the function of *BrAP2a* or *BnAP2* could result in a sepal–carpel modification phenotype. In *Prunus mume* (Rosaceae), a 49 bp deletion-mediated miR172 target site loss in AP2-like *PmAP2* (*APETALA2-like* gene) resulted in the transformation of stamens to petals [34]. However, mutation disrupting the miR172 target site of the orthologs of the TOE-type gene in peach and rose also resulted in the conversion of stamens to petals and even loss of floral determinacy (the double-flower trait) [35,36]. In the asterid species *Petunia*, the TOE-type gene *BEN* acts as a floral repressor and represses the C-function, while the AP2-type *ROB* genes are required for perianth and pistil development, as well as repressing the B-function (but not the C-function) together with *BEN* in the first floral whorl [11]. In the asterid species tomato (*Solanum lycopersicum*), the TOE-type gene *SlTOE1* regulates inflorescence development by repressing *STM3* and the tomato *SOC1* homolog *TM3* [37]. In carnation, a disruption of the miR172 target sequence of the TOE-type ortholog causes a dominant double-flower phenotype [38]. All these data indicate that the floral organ identity genes *AP2* or *TOE* orthologs show obvious functional diversity among core eudicots.

In the rosid species *A. thaliana*, *AP1* activates floral meristem identity (FMI) and specifies sepal and petal identities [39]. *AP1* down-regulates *ZP1* and *ZFP8* in floral meristems, lifting the repression on the class B (*AP3* and *PI*) and C (*AG*) genes, which leads to specific stamens and carpel identities [40]. In the asterid species *Petunia*, *AP1* orthologs are required for inflorescence meristem identity and act as B-function repressors in the first floral whorl, together with the AP2-type *ROB* genes [11,41]. All these suggest that *AP1* orthologs work together with AP2-type *ROB* genes involved in a perfect sepal development pathway in *Petunia*.

In common buckwheat, a small-scale gene duplication event occurs in *AP1* orthologs, resulting in *FaesAP1_1* and *FaesAP1_2* in *F. esculentum*. *FaesAP1_1* activates flowering and regulates tepal (perianth) development [24], while *FaesAP1_2* is mainly involved in filament length determination. In common buckwheat, both *FaesAP2* (AP2-type) and *FaesTOE* (TOE-type) are redundantly involved in style and/or filament length determination. Moreover, *FaesTOE* also seems to play a role in other developments. However, neither *FaesAP2* nor *FaesTOE* could directly repress the B and/or C class genes in common buckwheat. Interestingly, *FaesTOE* directly up-regulates *FaesAP1_2* to be involved in filament length determination in common buckwheat. Moreover, the knockdown of *FaesTOE* expression could result in expression down-regulation of the directly target *FaesAP1_2* in the *FaesTOE*-silenced LH plants. Our previous study also found another filament pathway in common buckwheat through *FaesAP3_1* (B-class gene) and *FaesELF3* [22]. However, how these genes work together to specify a perfect stamen still remains unclear. Our findings uncover a pathway controlling common buckwheat filament length and offer deeper insight into the functional evolution of *AP2* orthologs in the early-diverging core eudicots.

## 4. Materials and Methods

### 4.1. Plant Material

Long-homostyle (LH) common buckwheat lines with red/white flowers were grown under natural conditions in Jingzhou, Hubei Province, China. The roots, stems, juvenile leaves, tepals, stamens, pistils, and 6-day-old fruits (achenes) of LH plants were separately dissected, immediately frozen in liquid nitrogen, and stored at −80 °C until used. Moreover, the LH floral buds at sequential developmental stages were sampled. Each sample was divided in half; one was immediately frozen in liquid nitrogen and stored at −80 °C until used, and the other was fixed in FAA [38% formaldehyde: acetic acid: 70% ethanol = 1:1:18 (*v*/*v*)] for cytomorphological examination. *Nicotiana benthamiana* for the dual-luciferase reporter assay was planted in the growth chamber at 22 °C under long-day conditions (16 h light, 8 h dark).

### 4.2. Isolation and Characterization of APETALA2 Floral Homeotic Genes Long-Homostyle Common Buckwheat

Total RNA was extracted from LH floral buds using an EASYspin Plant RNA Kit (Aidlab, Hong Kong, China) according to the manufacturer’s protocol. The first-strand cDNA of 3′ RACE was prepared, and then the 3′ end cDNA sequences of *FaesAP2* and *FaesTOE* were separately amplified with gene-specific forward primers 3RGSPAP2 and 3RGSPTOE (Supplementary Table S1) using the 3-full RACE Core Set Ver. 2.0 kit (TaKaRa, Kusatsu, Japan) according to the manufacturer’s protocol, but with 90 s annealing at 58 °C. The forward primer design referenced the *AP2* homologous sequences (F01.PB24091 and F01.PB47570) identified before (BioProject ID: PRJNA517031). The phylogenetic tree was constructed using MEGA version 5.05 with the neighbor-joining (NJ) method. The NJ tree was constructed with 1000 bootstrap replications. All the APETALA2 homologous transcription factors (TF) with complete sequences were obtained from the NCBI Genbank (Supplementary Table S2).

### 4.3. Isolation and Sequence Analysis of FaesAP1_2 and FaesAP1_2 Promoter (pFaesAP1_2) from Long-Homostyle Common Buckwheat

Total RNA was extracted from LH floral buds, and the first-strand cDNA of 3′ RACE was prepared according to the protocol described above. The 3′ end cDNA sequence of *FaesAP1_2* was isolated with gene-specific primer 3RGSPAP1_2 (Supplementary Table S1) but with 60s annealing at 58 °C. The forward primer design referenced the *AP1* homologous sequence (F01.PB26508) identified before (BioProject ID: PRJNA517031). The phylogenetic analysis of *FaesAP1_2* was referenced to the method described before. The putative FaesAP1_2 protein sequence and other AP1/FUL-like proteins were selected for constructing phylogenetic trees obtained from the NCBI Genbank (Supplementary Table S3). In addition, the AGL6-like and SEP-like proteins were also included as outgroups because previous studies grouped them into the AP1/SEP/AGL6 superclade [41,42].

Buckwheat genomic DNA was extracted from LH plant juvenile leaves using the CTAB Plant Genomic DNA Rapid Extraction Kit (Aidlab, Beijing, China) according to the manufacturer’s protocol. The *FaesAP1_2* 5′ flanking region was isolated from LH buckwheat genomic DNA by using the Genome Walking Kit (TaKaRa, Japan) following the manufacturer’s protocol and with gene-specific primers DpAP1_2SP1, DpAP1_2SP2, and DpAP1_2SP3 (Supplementary Table S1) for the walking sequence. The putative transcription start site of *FaesAP1_2* was searched according to the methods described by Solovyev et al. [43], and the cis-acting elements located at the *FaesAP1_2* promoter region were searched in the PLACE database [44].

### 4.4. Yeast One-Hybrid Assay

A yeast one-hybrid (Y1H) assay was performed using the Yeast One-Hybrid Media Kit (Coolaber, Shanghai, China). The *pFaesAP1_1* (MK327554.1); *pFaesAP1_2*; *pFaesAP3_1* (MK956946.1); *pFaesAP3_2* (MN016952.1); *pFaesPI_1* (OM032614.1); *pFaesPI_2* (OM032615.1); *pFaesAG* and *pFaesELF3* (OP572282.1) region containing TTTGTT and/or AACAAA motifs were separately cloned into pAbAi plasmid to construct pBait-AbAi vectors with the gene-specific primer pairs Y1HpFaesAP1_1F and Y1HpFaesAP1_1R for *pFaesAP1_1* (−1171/−657), Y1HpFaesAP1_2F and Y1HpFaesAP1_2R for *pFaesAP1_2* (−450/+35), Y1HpFaesAP3_1F and Y1HpFaesAP3_1R for *pFaesAP3_1* (−622/+114), Y1HpFaesAP3_2F and Y1HpFaesAP3_2R for *pFaesAP3_2* (−272/+125), Y1HpFaesPI_1F and Y1HpFaesPI_1R for *pFaesPI_1* (−1289/−687), Y1HpFaesPI_2F and Y1HpFaesPI_2R for *pFaesPI_2* (−402/+31), Y1HpFaesAGF and Y1HpFaesAGR for *pFaesAG* (−1102/−692), as well as Y1HpFaesELF3F and Y1HpFaesELF3R for *pFaesELF3* (−24/+554) (Supplementary Table S1) [3]. Two common buckwheat *AP2*-like genes, *FaesAP2* and *FaesTOE*, cDNAs containing full-length ORFs (Open Reading Frame, ORF), were separately cloned into the pGADT7 plasmid to construct prey vectors with the gene-specific primer pairs Y1HFaesAP2F and Y1HFaesAP2R for *FaesAP2*, as well as Y1HFaesTOEF and Y1HFaesTOER for *FaesTOE* (Supplementary Table S1). The linearized plasmids pAbAi-*pFaesAP1_1*, pAbAi-*pFaesAP1_2*, pAbAi-*pFaesAP3_1*, pAbAi-*pFaesAP3_2*, pAbAi-*pFaesPI_1*, pAbAi-*pFaesPI_2*, pAbAi-*pFaesAG*, and pAbAi-*pFaesELF3* were separately transformed into the Y1H Gold-competent cells to generate bait-reporter strains. The transformants were selected on SD/-Ura media and screened for the minimal inhibitory concentration of Aureobasidin A (AbA) for the bait-reporter strains. Two prey plasmids, pGADT7-*FaesAP2* and pGADT7-*FaesTOE,* were separately transformed into the bait yeast strains for Y1H according to the manufacturer’s protocol. Colonies were incubated and selected on the medium (SD/-Leu/AbA) with the bait of minimum AbA resistance for 3 days at 30 °C.

### 4.5. Dual-Luciferase Reporter Assay

The *pFaesAP1_2* region was cloned into the pGrenen0800-LUC vector to generate the reporter plasmid pGreen0800-*pFaesAP1_2* with the gene-specific primer pairs Dual-pFaesAP1_2F and Dual-pFaesAP1_2R (Supplementary Table S1). The full-length ORF of *FaesTOE* was cloned into the pGreenII 62-SK vector to generate the effector vector with the gene-specific primer pairs Dual-FaesTOEF and Dual-FaesTOER (Supplementary Table S1). The reporter vector and effector vector were separately introduced into *Agrobacterium* strain GV3101 (pSoup). The effector and the reporter *Agrobacterium* cultures were mixed together at a ratio of 2:1 and infiltrated into *N. benthamiana* leaves. Empty pGreenII 62-SK was cotransformed with the pGreen0800-*pFaesAP1_2* reporter as a negative control. The treated tobacco plants were cultured in the dark for 24 h before being transferred to the growth chamber at 22 °C under long-day conditions (16 h light, 8 h dark) for 3 days. Firefly luciferase and renilla luciferase were assayed using the Duo-LiteTM Luciferase Assay System (Vazyme, Nanjing, China) following the manufacturer’s protocol. Each combination had three biological replicates.

### 4.6. Expression Analysis of FaesAP2 and FaesTOE

The total RNA of each sample was extracted according to the above method, but the first-strand cDNA was synthesized for quantitative real-time PCR (qRT-PCR) by using the HiScript^®^ II Q RT SuperMix for qPCR kit (Vazyme, Nanjing, China) following the manufacturer’s protocol. The relative expressions of *FaesAP2* and *FaesTOE* were separately detected in root, stem, juvenile leaf, tepal, stamen, pistil, and 6-day-old fruit of LH lines by qRT-PCR referring to Ma et al. [22], but with the gene-specific forward primer qFaesAP2F and reverse primer qFaesAP2R for *FaesAP2*, and with the gene-specific forward primer qFaesTOEF and reverse primer qFaesTOER for *FaesTOE*, respectively (Supplementary Table S1). In addition, *FaesAP2* and *FaesTOE* expressions were also detected in LH floral buds at sequential developmental stages with the qRT-PCR suggested above. The developmental stages of LH floral buds were confirmed in the paraffin section suggested by Ma et al. [22]. The amplicons of the *F. esculentum* actin gene (Genbank accession number: HQ398855.1) were amplified as the internal control with primer pairs qFaesactinF and qFaesactinR. The experiments were repeated three times for each sample.

### 4.7. VIGS Assay in Long-Homostyle Buckwheat

A 487 bp fragment of the *FaesTOE* cDNA, a 484 bp fragment of the *FaesAP2* cDNA, and a 381 bp fragment of the *FaesAP1_2* cDNA were separately cloned into the TRV2-mediated VIGS vectors with *XbaI* and *SacI* restriction enzymes, but with the gene-specific primer pairs TRV2-FaesTOEF and TRV2-FaesTOER for *FaesTOE*, TRV2-FaesAP2F and TRV2-FaesAP2R for *FaesAP2*, and TRV2-FaesAP1_2F and TRV2-FaesAP1_2R for *FaesAP1_2*, respectively.

Then, the TRV2-*FaesTOE*, TRV2-*FaesAP2*, TRV2-*FaesAP1_2*, TRV2, and TRV1 empty vector plasmids were separately introduced into *Agrobacterium* strain GV3101. The TRV-mediated VIGS vectors were infiltrated into the leaves of the two-true-leaves stage seedlings of LH lines mediated by *Agrobacterium tumefaciens* strain GV3101 with the method suggested by Liu et al. [45]. All the infected seedlings were cultivated in darkness for 24 h and then moved to the greenhouse at a temperature of 22 °C under short-day conditions. The expression levels of *FaesTOE*, *FaesAP2*, and *FaesAP1_2* were separately detected by qRT-PCR in the flowers of VIGS-silencing plants. Plants were treated for each construct to make sure that flowers with the strongest phenotypic changes were seen in at least three individuals.

## 5. Conclusions

Common buckwheat belongs to the order Caryophyllales (an early-diverging core eudicots clade) and produces distylous flowers with single-whorl showy undifferentiated perianth (tepals), representing an obvious difference with most core eudicots flowers. Here, we have developed a self-compatible (SC) common buckwheat line with uniform long-homostyle (LH) flowers that can easily fix useful agronomical traits, which makes it an excellent candidate line to explore the molecular mechanisms of floral development and evolution in the *Fagopyrum* genus. In this study, two *euAP2* orthologs, *FaesAP2* and *FaesTOE,* and an *AP1* orthologous gene, *FaesAP1_2,* were isolated and identified from LH common buckwheat. Moreover, Y1H was performed to screen whether *FaesTOE* or *FaesAP2* could directly regulate ABC MADS-box genes identified by us before. However, neither *FaesAP2* nor *FaesTOE* could directly repress the B and/or C class genes in common buckwheat. The Dual-Luciferase Reporter (DR) and VIGS Assay further suggested that *FaesTOE* directly activates *FaesAP1_2* (but not the C-functional gene *FaesAG*) to regulate filament length. Moreover, VIGS-silencing LH common buckwheat showed that *FaesAP2* is mainly involved in controlling filament and style length, but *FaesTOE* is mainly involved in regulating filament length and pollen grain development. Both the *FaesTOE* and *FaesAP2* genes redundantly function in regulating filament length determination in LH common buckwheat. In addition, the *FaesAP1_2* silenced flower showed tepal numbers, and filament length decreased obviously. Moreover, the knockdown of *FaesTOE* expression could result in expression down-regulation of the direct target *FaesAP1_2* in the *FaesTOE*-silenced LH plants. Our findings uncover a stamen development pathway in common buckwheat and offer deeper insight into the evolutionary history of the AP2 family.

## Data Availability

All data generated or analyzed during this study are included in this published article. Further inquiries can be directed to the corresponding author.

## Supplementary Materials

The following supporting information can be downloaded at: https://www.mdpi.com/article/10.3390/ijms25137193/s1.
