# Supplementary material for: APETALA2-like Floral Homeotic Protein Up-Regulating FaesAP1_2 Gene Involved in Floral Development in Long-Homostyle Common Buckwheat
Source: Int J Mol Sci. 2024 Jun 29;25(13):7193. doi: 10.3390/ijms25137193 (PMC11241573; doi:10.3390/ijms25137193)
Supplement: Supplementary file 1 [file ijms-25-07193-s001.zip › ijms-3045004-supplementary.pdf]

Supplementary Material

Supplementary Table S1: Primers used in this study.

Primer name	Primers sequences (5'-3')
3RAP2GSP	ATACAACAGGGAAACACAGCT
3RTOEGSP	GCAAGCTCTTTTCCCTTTAACAG
3RAP1_2GSP	GGCCACACAACTACACCAAAAATTG
DpAP1-2SP1	GATCTTCTCCATGCTTGAATCAGTAG
DpAP1-2SP2	GACAATAAGGGCAACTTCAGCATCAC
DpAP1-2SP3	GCTTTCTTCACTAAACCTCCTCTTCT
Y1HFaesTOEF	ATGGAGGCCAGTGAATTCGCAAGCTCTTTTCCCTTTAACAG
Y1HFaesTOER	ATTCATCTGCAGCTCGAGCTCCTCCTTAAACCTCACATCTCCAT
Y1HFaesAP2F	ATGGAGGCCAGTGAATTCATACAACAGGGAAACACAGC
Y1HFaesAP2R	ATTCATCTGCAGCTCGAGCTCAAGAGGATGATACCCATTGT
Y1HpAP1_1F	AAGCTTGAATTCGAGCTCACTATATACGTGCATTAAGCTGT
Y1HpAP1_1R	ACATGCCTCGAGGTCGACTGGTTCGTCTACACTTATTTGGG
Y1HpAP1_2F	AAGCTTGAATTCGAGCTCCTCATATCCACCCTAATGCAG
Y1HpAP1_2R	ACATGCCTCGAGGTCGACTGGTGTAGTTGTGTGGCCAAA
Y1HpFaesPI_1F	AAGCTTGAATTCGAGCTCCAACCCGAAGCAACTACAAG
Y1HpFaesPI_1R	ACATGCCTCGAGGTCGACGGCAAGAGGACGAGAAAAGG
Y1HpFaesPI_2F	AAGCTTGAATTCGAGCTCTCAGCTACACTACAAACTTCTT
Y1HpFaesPI_2R	ACATGCCTCGAGGTCGACATTGTAATGGAAGAACGAGA
Y1HpFaesAP3_1F	AAGCTTGAATTCGAGCTCGCAACAAATGAGGAAGAGTGGTA
Y1HpFaesAP3_1R	ACATGCCTCGAGGTCGACGCAATCTCTCTAGTTCTTCCCTTG
Y1HpFaesAP3_2F	AAGCTTGAATTCGAGCTCCATTTTGAGATGTTTGTGA
Y1HpFaesAP3_2R	ACATGCCTCGAGGTCGACCATAACAAAGTAGGAAGAAAA
Y1HpFaesAG-F	AAGCTTGAATTCGAGCTCAGCATGTCTTTAACTTGACCT
Y1HpFaesAG-R	ACATGCCTCGAGGTCGACACTGAGTTGGGTAAGTTAGGG
Y1HpFaesELF3F	AAGCTTGAATTCGAGCTCGGAAACAGGAAAATCTAGCAT
Y1HpFaesELF3R	ACATGCCTCGAGGTCGACTCAACCTCTCTTAACAATCCA
Dual-FaesTOEF	CGCGGTGGCGGCCGCTCTAGAGCAAGCTCTTTTCCCTTTAACAG
Dual-FaesTOER	AAGCTTGATATCGAATTCCTCCTTAAACCTCACATCTCCAT
Dual-pFaesAP1_2F	GGGCCCCCCCTCGAGGTCGACCTCATATCCACCCTAATGCAG
Dual-pFaesAP1_2R	CAGGAATTCGATATCAAGCTTTGGTGTAGTTGTGTGGCCAAA
qFaesAP2F	GGTAAAGATGCTGTCACAAAC
qFaesAP2R	TGTTTCCTTGGATTGAGCTAC
qFaesAP1_2F	CCATAGCTCAAGAAGTGCAG

qFaesAP1_2R	TTACCCGATGTTGAGAGAAG
qFaesTOEF	CCCAAGGAAGATCTTAGAGGT
qFaesTOER	CAATAGCAACCCTACCATAAT
qFaesactinF	ACCTTGCTGGACGTGACCTTAC
qFaesactinR	CCATCAGGAAGCTCATAGTTC
TRV2-FaesAP2F	GTTACCGAATTCTCTAGAGAACCATACTGCAACTTGAT
TRV2-FaesAP2R	CTCGAGACGCGTGAGCTCCATTCGAAGGTGAGGATGAG
TRV2-FaesTOEF	GTTACCGAATTCTCTAGATTGATGGCCCTACGAGTAAC
TRV2-FaesTOER	CTCGAGACGCGTGAGCTCCTGAAGCTGAGCCTGAGCGA
TRV2-FaesAP1_2F	GTTACCGAATTCTCTAGACAAAGAGAGGGAAAGATCCATAG
TRV2-FaesAP1_2R	CTCGAGACGCGTGAGCTCTTATTCGGAAACTCGTGCACTTC

Supplementary Table S2: The sequence information of APETALA2-like proteins used to construct phylogenetic tree was selected from NCBI Genbank.

Taxon and species		Protein name	Accession numbers	Lineage
Arecaceae	*Elaeis guineensis*	EgAP2	XP_010932035.1	AP2
Musaceae	*Musa acuminata*	MaAP2a	XP_009418515.1	AP2
	Musa troglodytarum	MutrAP2	URD92577.1	AP2
Zingiberaceae	*Zingiber officinale*	ZoTOE3	XP_042381587.1	AP2
Poaceae	*Triticum aestivum*	WANT-1	BAH57730.1	ANT
		TaTOE1-B1	ANW09544.1	TOE
	Oryza sativa	OsAP2	CAJ86049.1	AP2
		OsIDS1	Q84TB5.1	TOE
		SNB	Q8H443.1	TOE
		OsPLT7	XP_025879549.1	ANT
		OsPLT8	XP_025882595.1	ANT
		OsPLT9	XP_015629409.1	ANT
	Zea mays	ZmRAP2-7	ABR19870.1	TOE
		ZmAP2L	ABR19870.1	TOE
Paeoniaceae	*Paeonia lactiflora*	PlAP2	AGI61068.1	AP2
	Paeonia suffruticosa	PasuAP2	AEK33829	AP2
Vitaceae	*Vitis vinifera*	VvAP2	NP_001267881.1	AP2
Fabaceae	*Medicago truncatula*	MetrAP2	KEH31465.1	AP2
		MtANT1	XP_039688487.1	ANT
	Glycine max	GmTOE4b	XP_003542008.1	TOE
		GmTOE4a	XP_003547042.3	TOE
Rosaceae	*Malus domestica*	MaDoAP2	ADE41133.1	AP2
	Kerria japonica	KjAP2	AXR86367.1	AP2
	Rosa chinensis	RcAP2L	XP_024186592.1	TOE
		RcAP2	XP_024182693.1	AP2

	Prunus persica	PpTOE	XP_007205143.1	TOE
		PpANT	XP_020422785.1	ANT
		PpAP2	XP_007208004.1	AP2
	Prunus mume	PmAP2L	XP_008224982.1	TOE
		PmAP2	AJT39804.1	AP2
Fagaceae	*Castanea mollissima*	CmAP2	KAF3973429.1	AP2
Cucurbitaceae	*Cucumis sativus*	CsTOE3	XP_004141908.1	AP2
Sapindaceae	*Xanthoceras sorbifolium*	XsAP2	KAH7566610.1	AP2
Brassicaceae	*Arabidopsis thaliana*	AtAP2	AAC13770.1	AP2
		AtTOE1	AAC49773.1	TOE
		AtTOE2	NP_200820.3	TOE
		AtTOE3	NP_201519.1	TOE
		AtSMZ	NP_191059.2	TOE
		AtSNZ	NP_850313.2	TOE
		AtANT	AAB17364.1	ANT
		AIL1	Q1PFE1.1	ANT
		AIL5	Q6PQQ3.2	ANT
		AIL6	Q52QU2.1	ANT
		AIL7	Q6J9N8.2	ANT
	Brassica rapa	BrAIL6	XP_009122044.2	ANT
		BrAIL7	XP_033129837.1	ANT
		BrANT-1	XP_009138530.2	ANT
	Brassica napus	BnAIL6	WFR93999.1	ANT
Polygonaceae	*Fagopyrum esculentum*	FaesAP2	AKI81900.1	AP2
		FaesTOE	WWB03765	TOE
		FaesANT	WWB03766	ANT
Solanaceae	*Petunia hybrida*	PhROB1	AAD39439.1	AP2
		PhROB2	APG29273.1	AP2
		PhROB3	APG29274.1	AP2
		PhBEN	AAD39440.1	TOE
		PhBOB	APG29272.1	TOE
		PhANT	AHC98702.1	ANT
	Solanum tuberosum	SoyuAP2	CAR92295.1	TOE
	Solanum lycopersicum	SlTOE1	XP_004246701.1	TOE
Acanthaceae	*Andrographis paniculata*	AnpaTOE3	XP_051114135.1	TOE
Lamiaceae	*Salvia splendens*	SaspTOE3	XP_042016810.1	TOE

Supplementary Table S3: The sequence information of APETALA1-like proteins used to construct phylogenetic tree was selected from NCBI Genbank.

Taxon and species		Protein name	Accession numbers	Lineage
Orchidaceae	*Dendrobium hybrid cultivar*	DOAP1	ARI44760.1	Ful-Like
	Oncidium hybrid cultivar	OAP1	ADJ67240.1	Ful-Like
		OSEP3	ADJ67238.1	SEP
		OAGL6	ADJ67239.1	AGL6
Poaceae	*Brachypodium distachyon*	BdFUL2	ADQ92357.1	Ful-Like
	Oryza sativa	OsMADS6	Q6EU39.1	AGL6
Eupteleaceae	*Euptelea pleiosperma*	EpFUL-1	ABG49518.1	Ful-Like
		EpFUL-2	ABG49519.1	Ful-Like
		EuplSEP1	ADC79707.1	SEP
		EuplSEP3	ADC79706.1	SEP
Berberidaceae	*Epimedium sagittatum*	EsFUL	AEX58637.1	Ful-Like
		EsAGL2-1	AEX58639.1	SEP
		EsAGL2-2	AEX58640.1	SEP
		EsAGL6	AEX58638.1	AGL6
Ranunculaceae	*Nigella damascena*	NdFL2	ALM95511.1	Ful-Like
		NdFL1	ALM95510.1	Ful-Like
		NdSEP2	ALM95518.1	SEP
		NdSEP1	ALM95517.1	SEP
		NdAGL6	ALM95509.1	AGL6
	Aquilegia coerulea	AqFL1a	AGX01552.1	Ful-Like
		AqFL1b	AGX01553.1	Ful-Like
Cercidiphyllaceae	*Cercidiphyllum japonicum*	CejaFUL	ASY97766.1	AGL79
		CejaAGL6	ASY97761.1	AGL6
Saxifragaceae	*Heuchera americana*	HeaFUL	AAP83374.1	euFUL
Vitaceae	*Vitis vinifera*	VFUL-L	AAT07448.1	AGL79
Fabaceae	*Glycine max*	GmAP1a	XP_003547792.1	euAP1
		GmAP1c	XP_003516454.1	euAP1
		GmAP1d	XP_006574961.1	euAP1
		GmAP1b	XP_003531957.1	euAP1
Rosaceae	*Malus domestica*	MdAP1	BAH10867.1	euAP1
		MdAGL6	NP_001280892.1	AGL6
	Prunus persica	PperFUL	CAJ28929.1	euFUL
		PpAGL6-1	XP_020412713.1	AGL6
		PpAGL6-2	XP_007220094.2	AGL6
	Prunus dulcis	PdAGL6	BBG97451	AGL6
	Rosa chinensis	RcFUL	QIH54603.1	AGL79

Betulaceae	*Betula pendula*	BpMADS4	Q39400	AGL79
Passifloraceae	*Passiflora edulis*	PaedAP1	AQN67666.1	euAP1
		PeFUL	AQN67667.1	euFUL
		PaedSEP1	AET98846.1	SEP
Lythraceae	*Punica granatum*	PgSEP3a	WHA03959.1	SEP
		PgSEP3b	WHA03960.1	SEP
Malvaceae	*Gossypium hirsutum*	GhSEP1	AEL33631.1	SEP
Brassicaceae	*Arabidopsis thaliana*	AtAP1	CAA78909.1	euAP1
		AGL79	AAN52802.1	AGL79
		SEP1	P29382.2	SEP
		SEP2	P29384.1	SEP
		SEP3	NP_850953.1	SEP
		SEP4	P29383.2	SEP
		AGL6	AEC10582.1	AGL6
Polygonaceae	*Fagopyrum esculentum*	FaesAP1-1	AKI81897.1	euAP1
		FaesAP1-2	WWB03767	euAP1
Amaranthaceae	*Spinacia oleracea*	SpAP1-1	ACE75943.2	euAP1
		SpAP1-2	ACE75944.2	euAP1
Rubiaceae	*Coffea arabica*	CaAP1	AHW58038.1	euAP1
		CaFUL	AHW58040.1	euFUL
		CaSEP3	AHW58034.1	SEP
		CaSEP4	AHW58033.1	SEP
		CaAGL6	AHW58046.1	AGL6
Solanaceae	*Nicotiana tabacum*	NAP1-2	AAD01422.1	euAP1
		NFUL	ABF82231.1	euFUL
	Petunia x hybrida	Ph-FBP2	AAA86854.1	SEP
		Ph-FBP5	AAK21248.1	SEP
		Ph-FBP4	AAK21247.1	SEP
		pMADS4	BAA94287.1	AGL6
	Solanum lycopersicum	SlAGL6	NP_001348459.1	AGL6
Plantaginaceae	*Antirrhinum majus*	AmFUL	AAP83363.1	euFUL
		DEFH28	AAK72467.1	AGL79
Asteraceae	*Tagetes erecta*	TeAP1-1	QOJ53895.1	euAP1
		TeAP1-2	QOJ53896.1	euAP1
		TeFUL1	QOJ53897.1	euFUL
		TeFUL3	QOJ53899.1	euFUL
		TeFUL2	QOJ53898.1	AGL79
		TeSEP1	QOJ53900.1	SEP
		TeSEP4	QOJ53904.1	SEP
	Chrysanthemum x morifolium	CmAP1	AAO22979.1	euAP1
		CmeuFUL	AAO22981.1	euFUL

	CmAGL79	AAO22980.1	AGL79
Gerbera hybrid cultivar	GSQUA5	CAX65663.1	euFUL
	GSQUA6	CAX65664.1	euFUL
	GSQUA2	CAX65661.1	AGL79
	GRCD6	ASP44958.1	SEP
	GRCD8	ASP44960.1	SEP

Supplementary Figure S1:*FaesAP1_2* promoter sequence

The transcription start site (+1) is in bold and boxed. The start codon ATG is in bold and boxed. Putative cis-acting regulatory elements are in bold and underline.

> *pFaesAP1_2*

-593 GAAAAAAGAG AGTATATTTT AACTTTTAAA G**GTGA**T**TTTG TT**TAAATTAA CCTATTAAAT
GTGANTG10 TTTGTT/AACAAA motif

-533 AG**TTATTT**AT A**AGAAA**TAGT GGGACAAA**AG AAA**GATCAAG GAAAAGTAAC AGTAAGCTAG
TATABOX5 POLLEN1LELAT5 POLLEN1LELAT5

-473 TCAT**GTGA**GA CTGAGAGCTA CATCTCATAT CCACCCTAAT GCAGTAAAAT TCCGTACAGC
GTGANTG10

-413 TCAAACTCAC ATCAA**CCAAT** CAAAG**ACCGA C**AACGCACCA ACTCTACAA**A ACCAACC**CGG
CCAATBOX1 DRECRTCOREAT MYBPLANT

-353 TTTTCGTAAA ACCACTAATT CTCCAAACCC CATACTTTCA CT**CCTTTT**CA GAAAAA**GTGA**
PYRIMIDINEBOXOSRAMY1A GTGANTG10

-293 AAAATAAAAT TATAAAAAAA GCACATATAT TTCCA**TTATT T**CGTATGAGT ACCATAATAA
TATABOX5

-233 CTATTCCTAG TAAAAAATTT ACAAACATCA CTCTGAGTAA AAACCCTTGA AAAAACCGTA

-173 AGATATTTAT CTGTGCCTAC AGCAAAAGGA GGAATAGTAC TATGT**AACAA A**ATTCATTAT
TTTGTT/AACAAA motif

-113 TTTCTTT**CTT ATTTTTG**GGA ATTTGTAAAT AAAAAGT**TTA TTT**AAT**CAAT** GGGTTATTCT
CArG-BOX TATABOX5 CAATBOX1

+1

-53 TGTTTTTATT TTTTTTATAT TTTTTGTGTT ACTGATATTG C**TATAAAT**GC TAA**C**TTTGCT
TATABOX2

+8 TTCTTCTTTT GGCCACACAA CTACACCAAA AATTGAAGA**A GAAA**AGATAA G**AGAAA**AAT**A**
POLLEN1LELAT5 POLLEN1LELAT5

+68 **TG**
